# IQ in childhood and the metabolic syndrome in middle age: Extended follow-up of the 1946 British Birth Cohort Study

**DOI:** 10.1016/j.intell.2008.09.004

**Published:** 2009-11

**Authors:** Marcus Richards, Stephanie Black, Gita Mishra, Catharine R. Gale, Ian J. Deary, David G. Batty

**Affiliations:** a MRC Unit for Lifelong Health and Ageing, London, UK; b MRC National Survey of Health and Development, London, UK; c MRC Epidemiology Resource Centre, University of Southampton, UK; d MRC Centre for Cognitive Ageing and Cognitive Epidemiology, University of Edinburgh, UK; e Department of Psychology, University of Edinburgh, UK; f MRC Social and Public Health Sciences Unit, University of Glasgow, UK

**Keywords:** Childhood IO, Metabolic syndrome, 1946 birth cohort

## Abstract

IQ in early adulthood has been inversely associated with risk of the metabolic syndrome in midlife. We tested this association in the British 1946 birth cohort, which assessed IQ at age eight years and ascertained the metabolic syndrome at age 53 years based on modified (non-fasting blood) ATPIII criteria. Childhood IQ was inversely associated with risk of the metabolic syndrome, but this association was almost entirely mediated by educational attainment and achieved occupational social class. This may be consistent with a pattern where childhood IQ is strongly associated with outcomes that reflect neurological disorder, such as the degenerative dementias, but less so with common chronic physical diseases of ageing.

The positive association between education and health is well established. Education shapes access to resources, e.g. money, knowledge, power, prestige (Link, Phelan, Miech, & Westin, 2008). Better educational attainment can lead individuals to environments or the adoption of behaviors that protect against ill-health (Mirowsky & Ross, 2003), and minimize exposure to negative life events and chronic stressors (Pearlin, 1989). Some of these processes may be explained by individual differences in cognitive abilities (IQ), which partly determine educational attainment, social and occupational status and income (Deary, Strand, Smith, & Fernandes, 2007; Feinstein & Bynner, 2004; Kuh & Wadsworth, 1991; Kuh, Head, Hardy, & Wadsworth, 1997; Neisser et al., 1996; Richards & Sacker, 2003). IQ is also related to the acquisition of health-related behaviors, such as moderate alcohol consumption, avoidance of smoking, and adoption or maintenance of physical activity and healthy diet (Whalley & Deary, 2001), as recently confirmed by, for example, Batty, Deary, Schoon and Gale (2007a). Finally, cognition may be associated with health to the extent to which it is a marker of neural integrity (Whalley & Deary, 2001).

In regard to chronic physical diseases, or risk factors for these diseases, higher IQ scores are associated with lower levels of blood pressure or lower risk of hypertension (Batty, Deary, Schoon and Gale, 2007b; Lindgarde, Furu, & Ljung, 1987; Starr et al., 2004), cardiovascular disease (Hart et al., 2004) and obesity (Chandola, Deary, Blane, & Batty, 2006; Sorensen, Sonne-Holm, & Christensen, 1983; Teasdale, Sorensen, & Stunkard, 1992). The few reports of the relation of IQ with later measurement of serum cholesterol and high blood glucose or diabetes (Hart et al., 2004) do not find an association, although the studies in question were small. Some of these risk indices represent the constellation of factors referred to as the metabolic syndrome (Ford, Giles, & Dietz, 2002), which has an age-adjusted prevalence in the USA of 23.7% (43.5% for those aged 60 years and older). The relation between IQ and this condition has been recently examined in a cohort of former US Vietnam-era army personnel; scores from an IQ test administered on conscription at around 20 years of age were inversely related the metabolic syndrome and several of its components, as ascertained two decades later (Batty et al., 2008), an effect that was not explained by education. However, this cohort only comprised men, and only those with a rank of sergeant or below, which might have resulted in a narrowing of the SES range, leading to an underestimation of the relation between IQ and the metabolic syndrome. In addition, IQ was measured at age 20 years, after many participants had completed formal schooling (Deary et al., 2007; Neisser et al., 1996; Richards & Sacker, 2003). While childhood and early adult IQ are highly correlated it would be better to have a measure of mental ability before the completion of education, to minimize any effect of the latter on the former.

Data from the 1946 British Birth Cohort Study affords an excellent opportunity to examine the IQ-metabolic syndrome association in men and women in a large prospective population-based study that assessed IQ in childhood, ascertained a range of conditions and functions related to health, and characterized educational attainment and socio-economic position across the life course. Based on the findings of Batty et al. (2008), our study hypothesis was that cognition, measured in early childhood so as to be as free as possible from the influence of prior education and attained social position, would be inversely associated with risk of the metabolic syndrome.

## 1 Methods

### 1.1 Study participants

Participants were drawn from the on-going MRC National Survey of Health and Development (also known as the British 1946 birth cohort) that initially consisted of 5362 children (Wadsworth, Kuh, Richards, Hardy, 2006). At its inception, the sample comprised all births to non-manual and agricultural workers plus a random sample of one in four of manual workers selected from all single births within marriage that occurred in England, Wales and Scotland during one week in March, 1946. Information about socio-demographic factors and medical, cognitive and psychological function has been obtained regularly over the years by interview and examination, most recently in 1999, when 3035 study members at the age of 53 years underwent interview and examination by trained research nurses. Ethical approval for this research was obtained from the North Thames Multi-Centre Research Ethics Committee, and from relevant local research ethics committees in the survey areas. Informed consent was obtained from all participants.

### 1.2 Ascertainment of the metabolic syndrome

Measures taken at age 53 years during a home visit included the following: waist circumference measured at a point midway between the costal margin and the iliac crest and in line with the mid-axilla; brachial blood pressure measured twice in succession with the survey member sitting; and a non-fasting venous blood sample, from which HDL cholesterol, triglyceride and glycosylated hemoglobin (HbA1c) levels were assayed (for details of these assays see Langenberg, Kuh, Wadsworth, Brunner, & Hardy, 2006). We defined the metabolic syndrome and its components using a modified version of the Adult Treatment Panel III (ATPIII) recommended criteria (NCEP, 2001). According to this definition, participants were classified as having the metabolic syndrome if any three of the following were present: waist circumference above 102 cm for men or 88 cm for women; blood pressure ≥ 130/85 mmHg on second reading or use of antihypertensive medication; triglycerides ≥ 1.7 mmol/L; HDL cholesterol level < 1.036 mmol/L in men or 1.295 mmol/L in women; and HbA1c level in the top gender-specific quarter of the distribution (> 5.8% among men and women) or medication for diabetes. Modification of the ATPIII criteria was based on substitution of the latter for fasting plasma glucose, which was not available in this cohort (Langenberg et al., 2006).

### 1.3 Predictor variables

#### 1.3.1 Childhood IQ

Cognition was first assessed in this cohort at age eight years, using tests of verbal and nonverbal ability devised by the National Foundation for Educational Research (Pigeon, 1964). These tests were (1) Reading comprehension (selecting appropriate words to complete 35 sentences); (2) Word Reading (ability to read and pronounce 50 words); (3) Vocabulary (ability to explain the meaning of 50 words); and (4) Picture Intelligence, consisting of a 60-item non-verbal reasoning test. Scores from these tests were standardized to a mean of 0 and SD of 1, summed to create a total score representing overall cognitive ability at eight years, then re-standardized.

#### 1.3.2 Educational attainment

The highest educational or training qualification achieved by 26 years was classified by the 8-point Burnham scale (DES, 1972), recoded into no qualification, below ordinary secondary qualifications (vocational), ordinary secondary qualifications (‘O’ levels and their training equivalents), advanced secondary qualifications (‘A’ levels and their equivalents), or higher qualifications (degree or equivalent).

#### 1.3.3 Occupational social class at 43 years

Occupation of head of household at age 43 years was assigned according to the Registrar General (RG) system (OPCS, 1970), classified as professional (I), managerial/intermediate (II), skilled non-manual (IIInm), skilled manual (IIIm), semi-skilled manual (VI), and unskilled (V).

### 1.4 Statistical methods

#### 1.4.1 Regression

Logistic regression was used as an initial test of associations between childhood cognitive function, entered as a continuous variable, and the metabolic syndrome and its individual components. As a first step IQ × sex interactions were tested. Then, initially unadjusted associations were adjusted for educational attainment or adult social class, both entered as categorical variables.

#### 1.4.2 Structural equation model

Structural equation modeling (SEM) was used to describe the association, if any, of childhood IQ, educational attainment and adult occupation with the metabolic syndrome. This model contained three components: 1) paths from childhood cognitive function to education, own occupation and the metabolic syndrome; 2) paths from education and occupation to the metabolic syndrome (and the internal path from education to occupation; 3) paths from the individual components of the metabolic syndrome. Following the study of Batty et al. the explicit hypothesis tested here was that cognitive ability at age 8 years influenced the latent metabolic syndrome trait, and that some of that effect was mediated via education and social class, close correlates of IQ. The fit of the model was tested comprehensively, as described in the results section.

#### 1.4.3 Estimating the model

The analyses were all carried out using the latent variable modeling program Mplus version 5 (Muthén and Muthén, 2004). The estimation method used was the robust weighted least squares estimator (WLSMV) for categorical outcomes, which handle missing data by using pairwise present. This method is preferable to estimation based on complete data, i.e. the list-wise deletion (LD) approach, since estimates using missing data tend to be less biased and more reliable, even when the data deviate from missing at random and are non-ignorable (Arbuckle, 1996). Sample size was 1799 after LD on all variables in the model, and 4092 after incorporating missing data.

Two criteria were used to assess the fit of the model to the data. The root mean square error of approximation (RMSEA) (Steiger, 1990) gives a measure of the discrepancy in fit per degrees of freedom. It is bounded below by zero, only taking this value if the model fits exactly. If the RMSEA is < 0.05, the model is considered a close fit to the data. The final index of choice is the comparative fit index (CFI) (Bentler, 1990) whose values are restricted to lie on a 0 to 1 continuum, with higher values indicating a better fit. The CFI of a model is normally tested against a minimum criterion value of 0.95. We did not use the *χ*^2^ statistic since this is overly sensitive to model misspecification when sample sizes are large.

## 2 Results

Those with missing data for metabolic syndrome classification at 53 years had lower cognitive scores at age 8 years (*p* < 0.001), lower educational attainment (*p* < 0.001 for no vs. any qualifications) and lower occupational social class (*p* = 0.007 for manual vs. non-manual) than those who were able to be classified with or without the outcome. Table 1 shows descriptive statistics for the predictor variables by presence or absence of the metabolic syndrome. Around one fifth of the study participants were positive for the syndrome based on the definition used. Those with the syndrome had lower cognitive scores at age 8 years, and had lower educational and occupational attainment than those without the syndrome.

Preliminary logistic regression showed a main effect of sex (OR = 0.74, 95% CI = 0.59–0.93, *p* = 0.009) with childhood cognition as the only other independent variable in the model, although the cognition by sex interaction term was not significant (*p* = 0.95). On this basis analyses were not stratified by sex, but, to be consistent with the study of Batty et al. (2008) were also not adjusted for sex. Childhood cognition was inversely associated with risk of the metabolic syndrome (OR_one SD increase in IQ score_ = 0.86, 95% CI = 0.76–0.96, *p* = 0.008), close to the age-adjusted odds reported by Batty et al., 2008 (0.86, 95% CI = 0.80–0.99). This association was attenuated, and no longer significant at the 5% level, after adding educational attainment (OR = 0.96, 95% CI = 0.94–1.11, *p* = 0.60), or adult social class (OR = 0.93, 95% CI = 0.82–1.05, *p* = 0.23). Both latter variables were themselves inversely associated with risk of the metabolic syndrome (*p* for trend =0.03 for both). Childhood cognition was inversely associated with risk of each of the five components of the metabolic syndrome, with approximately equal magnitude (OR range = 0.85–0.92), although only associations with central obesity, hypertension and triglyceride level were significant at the 5% level. These latter associations were again attenuated by education and adult social class. Correlations between IQ, education and occupational social class were 0.57 between IQ and education, and 0.51 between education and occupation (both *p* < 0.001).

Un-standardized and standardized estimates from the SEM model were then calculated, for the available complete sample data (LD) and in the presence of missing data. Since results from these were similar, we here present results only from the latter.

**Fig. 1:**
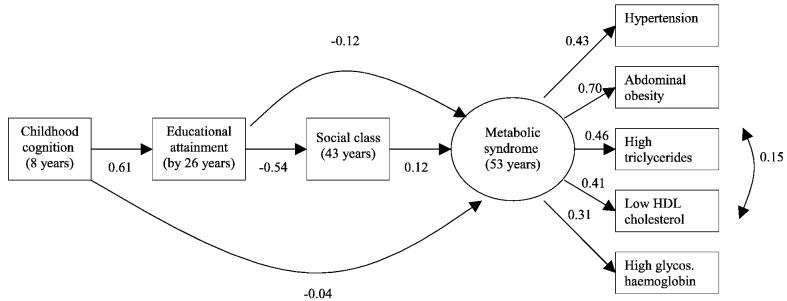
Standardized regression weights representing pathways between childhood cognition, educational attainment, adult occupational social class, and the metabolic syndrome (*n* = 4092; CFI = 0.97; RMSEA = 0.029).

Fig. 1 shows the SEM model. The numerical values refer to standardized estimates. Since RG social class is inversely coded (higher values for lower occupational attainment), positive estimates for this variable represent a risk effect on the outcome. Goodness of fit statistics indicated that the model was an adequate representation of the data (CFI = 0.97; RMSEA = 0.029). Unstandardized estimates for all paths were statistically significant at the 5% level except the direct path from childhood cognition to the metabolic syndrome (*p* = 0.29). As already noted, the direct path between childhood cognition and the metabolic syndrome was of negligible magnitude, and not significant at the 5% level. Direct paths between education and the metabolic syndrome, and between occupation and the metabolic syndrome, were of modest strength and of similar magnitude to each other (as noted, the positive value for occupation represents a risk effect).

As expected, there were strong direct paths from cognitive ability to educational attainment, and from educational attainment to own occupation.

Total standardized indirect effects of childhood IQ on the outcome were − 0.12, although specific indirect effects of childhood cognition via education (− 0.08), and via education and occupation (− 0.04) were of smaller magnitude.

## 3 Discussion

The main finding of this study was that childhood IQ showed an inverse association with the metabolic syndrome in midlife, an association that was almost entirely mediated by educational and occupational attainment.

Three study limitations should be noted. First, the definition of the metabolic syndrome was based on assays from non-fasting blood. This may cloud the interpretation of triglyceride levels (although not HDL cholesterol) and does not allow a standard measure of diabetes or glucose resistance (Langenberg et al., 2006). Second, there was a disproportionate loss to follow-up of those who were socially disadvantaged, although our SEM model was able to take account of missing data, and there is in any case no reason to believe that these survey members had a different metabolic profile to those of similar disadvantage who were assessed at 53 years (Langenberg et al., 2006). Third, our SEM model was deliberately simplified for ease of interpretation, although there are other important pathways to be investigated in this context, particularly the possible mediating effect of health behaviors such as diet, smoking and exercise, some of which are predicted by IQ (e.g. Batty et al., 2007a). As a counter-weight to these limitations our study design has a number of important strengths, including a nationally-representative sampling frame, and a measure of IQ that preceded ascertainment of the metabolic syndrome by several decades, ruling out the possibility of reverse causality where components of the metabolic syndrome themselves could have lead to a reduction in cognitive function (MacLullich, Deary, Starr, Walker, & Seckl, 2004). Of equal importance, as a relatively young cohort the NSHD is less prone to “healthy survivor” bias from selective mortality than samples in later life.

With these strengths and limitations in mind, how should the lack of a direct association between childhood cognition and the metabolic syndrome be interpreted? To begin with, the results of this study differ from those of Batty et al. (2008) in the Vietnam Experience Study. While the latter found an association between early adult IQ and the metabolic syndrome in men in midlife that was similar in magnitude to the total indirect effects of childhood IQ on this outcome in the 1946 cohort (0.13 and 0.12, respectively), the association in the Vietnam study was robust to adjustment for education. This discrepancy is unlikely to have resulted from the different cognitive tests in these two studies, in view of independent evidence that general (*g*) factors identified in different cognitive test batteries were almost perfectly correlated in the same individuals (Johnson, Bouchard, Krueger, McGue, & Gottesman, 2004; Johnson, Nijenhuis, & Bouchard, 2008). This strongly suggests that *g* is robust to its particular test score ingredients. A more likely possibility is that, while childhood IQ is strongly associated with educational attainment (Deary et al., 2007; Richards & Sacker, 2003), cognitive ability receives input from education that is independent of prior IQ (Richards & Sacker, 2003). Thus educational attainment may add little variance to an outcome over and above IQ if IQ is measured around the same time as, or after, formal education was completed, as in the Vietnam veterans study. In contrast, the measure of IQ used in the present study, obtained seven years before the minimum school leaving age, was relatively (although not of course entirely) free of the influence of education. By this logic cognitive ability that is proximal to an adult health outcome should, as a measure that represents an accumulation of influence across the life course (Richards & Deary, 2005) be more predictive of that outcome than cognition that is more distal (although, as noted, the more proximal cognition is to the outcome the greater the challenge of reverse causality). An exception is where childhood cognition may better reflect critical influences in the early life course than later cognition, which we have suggested is the case for timing of the natural menopause in relation to early estrogenic programming (Richards, Kuh, Hardy, & Wadsworth, 1999).

This in fact raises a broader issue, namely the range of health outcomes that IQ might independently predict. Given that cognition reflects neural function, it is not surprising that it is predictive if the outcome is dementia risk. This is indeed the case, whether IQ is assessed in childhood (Whalley et al., 2000), estimated from complexity of biographical information written in early adulthood (Snowdon et al., 1996), or represented by the NART in later life (Schmand, Smit, Geerlings, & Lindeboom, 1997). Independent associations from IQ are also observed for other health outcomes that are closely related to neural function. These include timing of the natural menopause, as noted above (with implications for several physical health outcomes; Richards et al., 1999), and mental health (Batty, Mortensen, & Osler, 2005; Hatch et al., 2007; van Os, Jones, Lewis, Wadsworth, & Murray, 1997). On the other hand IQ may play a contributory but not dominant role when the outcomes are chronic physical diseases (Richards 2007), such as obesity or those of the cardio-pulmonary systems. Evidence for this suggestion is mixed.

Childhood IQ is associated with midlife FEV_1_ (Deary, Whalley, Batty, & Starr, 2006), after controlling for a range of confounders and mediators, including own education and occupation (Richards et al., 2005). However, preliminary evidence suggests that childhood IQ does not independently predict self-reported lung disease at this stage of the life course (Richards, 2007). In the Scottish Midspan studies linked to childhood IQ assessed as part of the Scottish Mental Survey l932, IQ was associated with measured blood pressure (Starr et al., 2004), and with diagnosed cardiovascular disease as a cause of death (Hart et al., 2004), after controlling for a range of potential confounders and mediators, including adult social class (although not education). Associations between childhood IQ and hypertension were not statistically significant in the Aberdeen Children of 1950s study (Batty, Deary, & MacIntyre, 2007c). Results in the British 1970 birth cohort (Batty et al., 2007b) were more equivocal. The odds ratio of high blood pressure per SD increase in mental ability at age 10 years were 0.90, which was attenuated only very slightly to 0.91 after adjustment for adult social class, and to 0.93 and no longer significant at the 5% level after adjustment for academic/vocational qualifications. While associations between childhood IQ and obesity were generally robust to confounders in the Aberdeen Children of 1950s study (Batty et al., 2007c), and the British 1970 (Batty et al., 2007b) and 1958 (Chandola et al., 2006) birth cohorts, they were largely explained by mediation via education (although there was an additional influence of healthy diet via IQ in the latter), consistent with the findings of Lawlor, Clark, Davey Smith, & Leon (2006) based on the Aberdeen Children of 1950s study. Consistent with the lack of association between childhood IQ and glycosylated hemoglobin in the present analyses, none of these studies found a significant association between childhood IQ and diabetes. A significant association between childhood IQ and self-reported diabetes was found in the British 1958 birth cohort by Olsson, Hulting, & Montgomery (2008), although in a separate study of the same cohort this was mediated by educational attainment and current social class (Batty, Gale, Chandola, & Deary, 2007d). It should be noted that it is still unclear to what extent mediation in this context represents the indirect effect of IQ, or aspects of education or socioeconomic status that are themselves genuinely causal with respect to physical health outcomes. Further mechanistic studies are required to resolve this issue.

In conclusion, while childhood IQ has been associated with a wide range of health outcomes, its association with the metabolic syndrome may be very small, even after allowing for its indirect effects through educational and occupational attainment. This may be consistent with a larger pattern, where childhood IQ is strongly associated with health-related outcomes that closely reflect neural function, such as the degenerative dementias and mental health, but less so with common chronic physical diseases of ageing. Further studies are required, however, before this conclusion can be accepted with confidence.

## Figures and Tables

**Table 1:** Descriptive statistics for the predictor variables by presence vs. absence of the metabolic syndrome (based on list-wise deletion, *n* = 1799).

Variable	No syndrome	Yes syndrome
*N*	1419 (78.9%)	380 (21.1%)
Childhood cognition (mean and SD)	22.79 (6.81)	21.77 (7.04)
Educational qualifications by 26 years		
No qualifications	463 (32.6%)	165 (43.4%)
Vocational only	115 (8.1%)	29 (7.6%)
Up to ‘O’ level	312 (22.0%)	68 (17.9%)
Up to ‘A’ level	373 (26.3%)	91 (23.9%)
Higher	156 (11.0%)	27 (7.1%)
RG occupational social class at 43 years		
I (professional)	151 (10.6%)	29 (7.6%)
II (managerial/intermediate)	617 (43.5%)	142 (37.4%)
IIInm (skilled non-manual)	163 (11.5%)	36 (9.5%)
IIIm (skilled manual)	356 (25.1%)	122 (32.1%)
IV (semi-skilled)	106 (7.5%)	37 (9.7%)
V (unskilled)	26 (1.8%)	14 (3.7%)
